# Diet standardization reduces intra-individual microbiome variation

**DOI:** 10.1080/19490976.2022.2149047

**Published:** 2022-11-25

**Authors:** Clara Delaroque, Gary D. Wu, Charlene Compher, Josephine Ni, Lindsey Albenberg, Qing Liu, Yuan Tian, Andrew D. Patterson, James D. Lewis, Andrew T. Gewirtz, Benoit Chassaing

**Affiliations:** a INSERM U1016, Team “Mucosal Microbiota in Chronic Inflammatory Diseases”, CNRS UMR 8104, Université Paris Cité, Paris, France; b Division of Gastroenterology and Hepatology, Department of Medicine, Perelman School of Medicine, University of Pennsylvania, Philadelphia, Pennsylvania, USA; c School of Nursing, University of Pennsylvania, Philadelphia, Pennsylvania, USA; d Division of Gastroenterology, Hepatology, and Nutrition, Department of Pediatrics, Children’s Hospital of Philadelphia, Philadelphia, Pennsylvania, USA; e Center for Molecular Toxicology and Carcinogenesis, Department of Veterinary and Biomedical Sciences, Pennsylvania State University, Philadelphia, Pennsylvania, USA; f Center for Clinical Epidemiology and Biostatistics, Perelman School of Medicine, University of Pennsylvania, Philadelphia, PA 19104, USA; Division of Gastroenterology and Hepatology, Department of Medicine, Perelman School of Medicine, University of Pennsylvania, Philadelphia, Pennsylvania, USA; g Institute for Biomedical Sciences, Center for Inflammation, Immunity and Infection, Digestive Disease Research Group, Georgia State University, Atlanta, Georgia, USA

**Keywords:** Intestinal microbiota, diet, microbiota stability

## Abstract

The human gut microbiota is highly heterogenous between individuals and also exhibits considerable day-to-day variation within individuals. We hypothesized that diet contributed to such inter- and/or intra-individual variance. Hence, we investigated the extent to which diet normalization impacted microbiota heterogeneity. We leveraged the control arm of our recently reported controlled-feeding study in which nine healthy individuals consumed a standardized additive-free diet for 10 days. Diet normalization did not impact inter-individual differences but reduced the extent of intra-individual day-to-day variation in fecal microbiota composition. Such decreased heterogeneity reflected individual-specific enrichment and depletion of an array of taxa microbiota members and was paralleled by a trend toward reduced intra-individual variance in fecal LPS and flagellin, which, collectively, reflect microbiota’s pro-inflammatory potential. Yet, the microbiota of some subjects did not change significantly over the course of the study, suggesting heterogeneity in microbiota resilience to dietary stress or that baseline diets of some subjects were perhaps similar to our study’s standardized diet. Collectively, our results indicate that short-term diet heterogeneity contributes to day-to-day intra-individual microbiota composition variance.

## Introduction

An extensive body of literature demonstrates the ability of a broad range of dietary components to rapidly alter the murine gut microbiome, resulting in an altered susceptibility to an array of inflammatory diseases.^1,2^ In contrast, the extent to which diet alters the human microbiome and, concomitantly, proneness to disease is much less clear. One factor contributing to such lack of clarity is the high degree of microbiota heterogeneity of humans both in terms of baseline differences in microbiota composition between individuals and lesser but, nonetheless, considerable degree of intra-individual variance when individuals are sampled on different days.^3,4^ Investigating how dietary components impact the microbiota of humans is also inherently complicated by the high degree of inter- and intra-individual dietary heterogeneity, thus potentially altering microbiota independent of the specific dietary component being studied, ultimately obscuring its effects.^5–8^ For these reasons, our recent clinical study of the common food additive carboxymethylcellulose (CMC), named Functional Research on Emulsifiers in Humans (FRESH),^9^ utilized a “each subject as their own control” design wherein the microbiota of each subject was monitored before, during, and after controlled feeding of a standardized additive-free processed food-free controlled diet (AF-PFF diet) enriched, or not with CMC. Our results accorded with our previous results in mice,^10^ with the observation that CMC altered microbiota while the extent of the observed impact was highly individual-specific.

The goal of the present study was to leverage the control arm of the FRESH study to characterize the extent to which diet normalization with an additive free diet might reduce inter- and/or intra-individual microbiota variance and moreover probe whether the resilience of the microbiota to perturbation by CMC might reflect a general trend that the human microbiota is relatively resistant to variety of dietary manipulations, including AF-PFF. We found that, indeed, the impact of AF-PFF on gut microbiota is highly personalized and did not alter the extent of inter-individual differences in composition or function. Nonetheless, diet standardization with AF-PFF reduced intra-individual variance, supporting the notion that diet heterogeneity contributes to microbiota variation. Moreover, our results suggest that studies that standardize diet may have increased sensitivity to detect alterations in microbiota that may be induced by a particular substance of interest.

## Results

### Inter-individual differences in microbiota composition dwarf potential impacts of diet standardization

**Figure 1.**
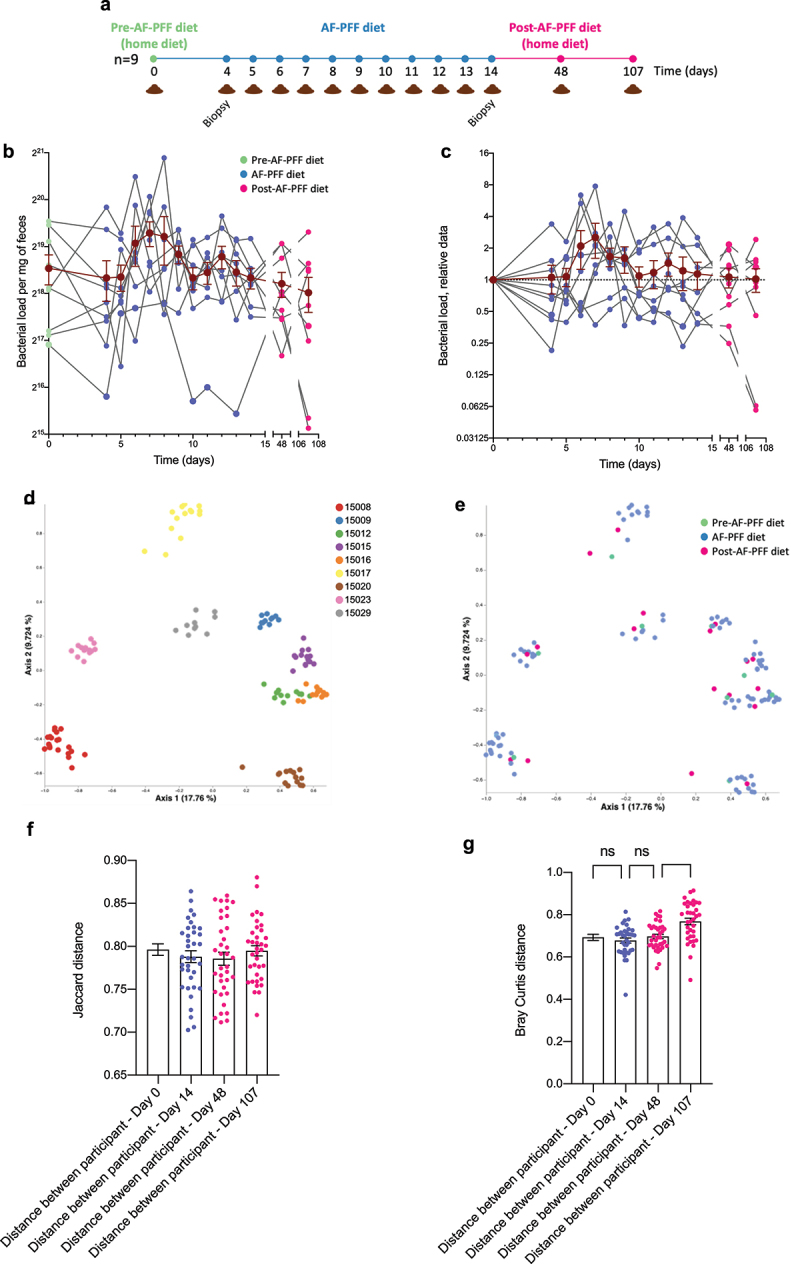
Inter-individual differences dominate impact of diet standardization on bacterial load and microbiota composition. (a) 9 healthy volunteers were subjected to a controlled diet between day 4 and day 14. (b-c) Individual fecal bacterial load through time expressed in bacteria number per g of feces (b), with data in (c) being normalized to 1 for day 0 samples in order to normalize for pre-intervention inter-individual variations. The dark brown line represents the mean ± SEM. (d-e) Principal Coordinate Analysis (PCoA) of the Jaccard distance matrix of the participants’ fecal microbiota assessed by 16S rRNA gene sequencing. All time points are included in the representation, and samples are colored by participants (d), or by timepoint categories (pre-controlled diet, during the controlled diet, and post-controlled diet) (e). (f) Jaccard distance between participant at day 0, 14, 48 and 107 of the study. (g) Bray-Curtis distance between participant at day 0, 14, 48 and 107 of the study. Mean ± SEM are represented and significance was assessed using one-way ANOVA with or without repeated measures corrected with a Dunnett posttest or Sidak posttest, respectively. (**p ≤ 0,01; n.s. indicates nonsignificant differences).

To discern the extent to which inter-individual and intra-individual (i.e., temporal) variability in microbiota was impacted by diet standardization, we took advantage of samples from the control arm of our recently reported study in which subjects (N = 9) were fed, under carefully controlled conditions, an additive-free processed food-free (AF-PFF) controlled diet for an 11-day period (Figure 1a). Fecal samples collected during, prior to, and following AF-PFF were used for microbiota assessment. The total level of bacteria per gm of feces, i.e. fecal bacterial density, was not significantly affected by AF-PFF diet consumption, albeit a non-statistically significant increase was observed during the first half of the AF-PFF diet feeding (Figure 1b, c). Principal coordinate analysis of the Jaccard beta diversity distance for all samples collected from the 9 participants demonstrated, as expected and as previously reported,^5,11^ a high level of variation in microbiota composition between participants, as illustrated by clear clustering of all samples collected from a given participant (Figure 1d). In contrast, coloring the plot based on the trial phase (pre, during or post AF-PFF diet) failed to show a major impact of diet phase on microbiota composition, indicating that any impacts of AF-PFF were modest relative to the extent of baseline inter-individual differences (Figure 1e). Accordingly, as presented figure 1f, inter-individual variations in microbiota composition were not dampened by a homogenous AF-PFF diet consumption in that the Jaccard distance observed between participants was similar under home (both pre- and post-intervention) and AF-PFF diets. Similar observations were made when analyzing Bray-Curtis distances (Figure 1g). Thus, diet standardization with an AF-PFF diet was not sufficient to reduce inter-individual variance in microbiota composition.

### AF-PFF diet standardization reduced day-to-day intra-individual microbiota compositional variation

**Figure 2.**
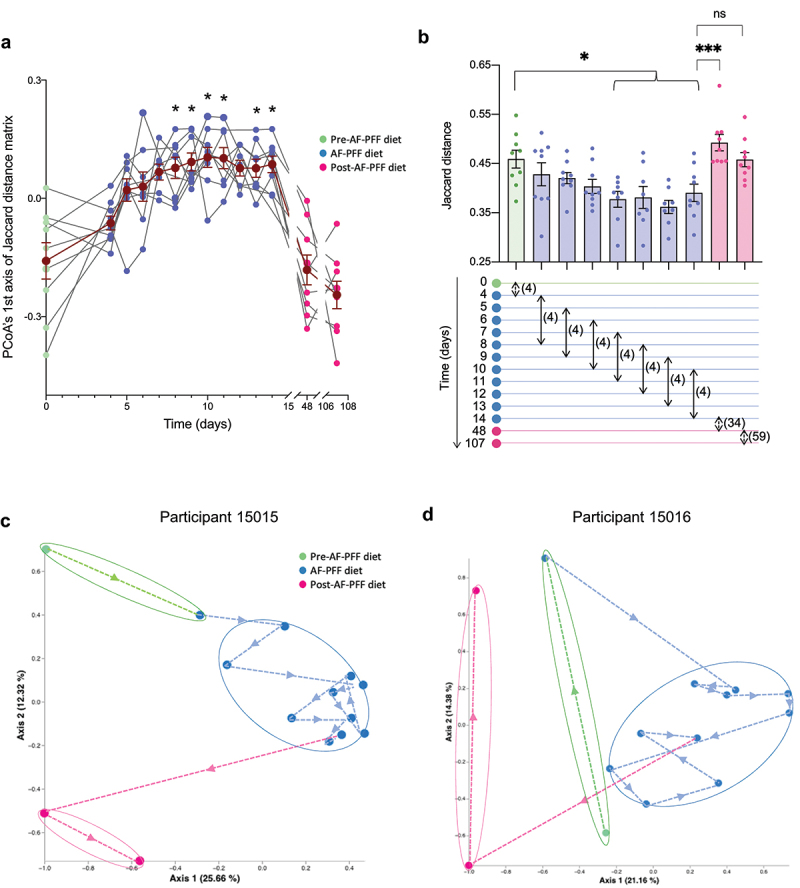
Diet standardization reduces extent of day-to-day changes in individuals’ microbiota composition. 9 healthy volunteers were subjected to a controlled diet between day 4 and day 14. (a) First axis of the PCoA of the Jaccard distance matrix of the participants’ fecal microbiota assessed by 16S rRNA gene sequencing. The dark brown line represents the mean ± SEM. (b) Jaccard distance within individuals – between day 0 (home diet) and day 4 (additive-free diet), between all 4 days apart within the additive-free diet, as indicated by arrows below the corresponding bar, between day 14 (during additive-free diet) and day 48 (home diet) and between day 48 and day 107 (home diet versus home diet). Numbers in brackets next to arrows indicate number of days separating timepoints analyzed. Each dot represents one participant. (c-d) PCoA of the Jaccard distance matrix of two representative participants. Each dot represents one time point, each color represents one phase of the study. Mean ± SEM are represented and significance was determined using one-way ANOVA with or without repeated measures, corrected for multiple comparisons with a Dunnett or Sidak posttest respectively. (*p ≤ 0,05; ***p ≤ 0,001; n.s. indicates nonsignificant differences).

We next analyzed the impact of AF-PFF diet on day-to-day variation in microbiota composition within participant. Intra-individual variation in microbiota composition was assessed *via* measuring daily changes in Jaccard distances when analyzing each participant independently. Such an approach demonstrated that inter-individual microbiota composition variations are significantly higher compared to intra-individual microbiota composition variations (**Fig. S1**), even when measured between home diet and AF-PFF diet. More importantly, visualizing day-to-day changes in microbiota composition by this approach revealed that microbiota of each subject appeared more stable during the AF-PFF diet phase compared to the pre- and post-intervention diet phases (Figure 2a). Accordingly, Jaccard distances separating same participant samples collected 4 days apart gradually decreased during the study, starting at 0,459± 0,018 and reaching 0,391 ± 0,018 at the end of the AF-PFF phase, further highlighting microbiota stabilization induced by consumption of a homogeneous AF-PFF diet (Figure 2b). Moreover, such inter-individual variations were significantly increased between the AF-PFF and the post-diet phases (0,493± 0,017), as well as when comparing, for each participant, two samples within the post-AF-PFF treatment phase (0,458 ± 0,015, Figure 2b). Altogether, these data suggest that day-to-day variations in microbiota composition are higher when on a heterogeneous home diet compared to when on a homogeneous AF-PFF diet.

Such notion was also evident when plotting Jaccard coordinates of representative participants, in which the extent of day-to-day changes during the AF-PFF diet phase (blue dots) were smaller than those observed during the pre- (green dots) or post- (pink dots) diet phases (Figure 2c-d). These observations indicate that daily feeding of the AF-PFF diet reduced day-to-day intra-individual variance in microbiota composition.

### AF-PFF diet induced targeted and individualized modulation of microbiota composition and function

**Figure 3.**
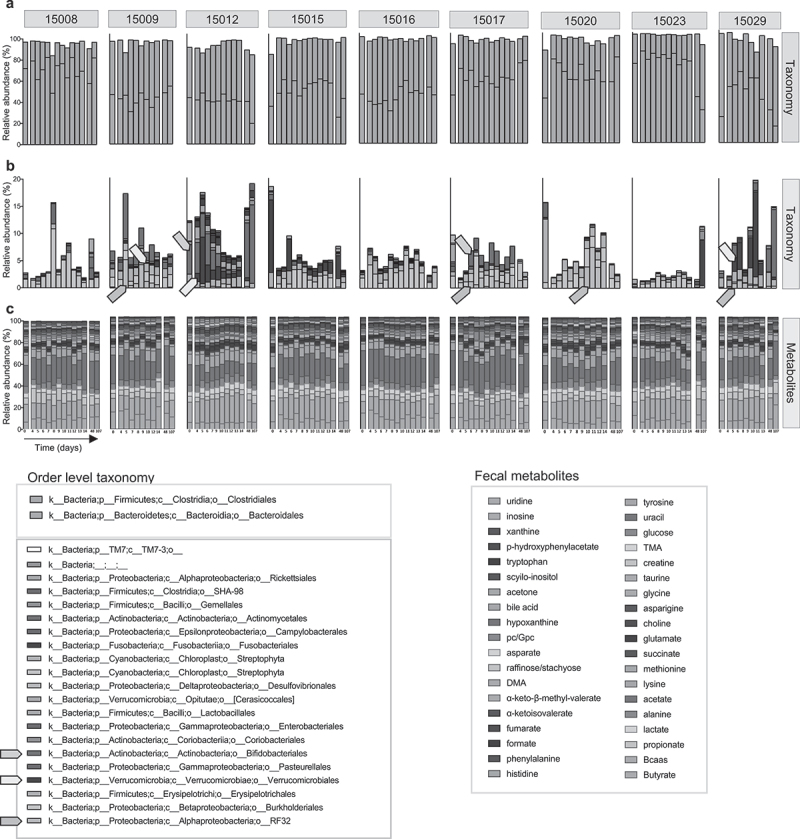
Diet standardization alters various microbiota members in select participants. 9 healthy volunteers were subjected to a controlled diet between day 4 and day 14. (a-b) Microbiota composition at the order taxonomic level are represented as relative abundance. *Clostridiales* and *Bacteroidales* orders are presented on panel a, and other orders are presented panelb, from day 0 to subsequent days. Arrows highlight microbiota orders discussed in results section. (c) Fecal metabolites relative abundance are presented for each participants from day 0 to subsequent days.

In order to better understand how diet standardization with AF-PFF stabilized intra-individual beta diversity, we next analyzed individual microbiota taxa at the order phylogenetic level. We observed that the AF-PFF diet impacted microbiota taxa in a highly individualized manner (Figure 3a, b). For example, participants 15009, 15017, 15020, and 15029 presented a stark increase in the *RF32* order, member of the proteobacteria phylum, upon AF-PFF diet consumption (Figure 3), which was not maintained following cessation of the diet standardization. Other examples include the *Bifidobacteriales* order in participants 15012 or 15017 and the *Verrucomicrobiales* order in participants 15009, 15012, and 15029 (Figure 3b). Such individualized changes in the abundance of microbiota members were not associated with stark changes in fecal metabolite profiles assessed through targeted metabolomic (Figure 3c).

Tracking individual microbiota members over time for all the participants highlighted the highly individualized response to AF-PFF diet (**Fig. S2**), in which select participants harbored a stark increase or decrease for a given microbiota member, which remained fully unaffected in other participants (**Fig. S2**). Similarly, most of the metabolites impacted by an AF-PFF diet consumption were participant-specific, while increase in select metabolites, including alanine, tyrosine, and branched chain amino acid, were observed for almost all participants during the AF-PFF diet phase (**Fig. S3**). Accordingly, computing metabolomic data through principal component analysis of the Bray Curtis distance demonstrated some level clustering per participants (**Fig. S4A**), while the impact of diet homogenization was not sufficient to normalize these differences (**Fig. S4A, C**) nor to impact intra-individual variations (**Fig. S4B, D**).

Thus, functional impact of an AF-PFF diet feeding period on the intestinal environment appears limited during this relatively short period, with highly individualized effects being observed. These observations suggest relatively high level of resilience in the intestinal microbiota composition and production of metabolites to dietary modulation and food additive withdrawal.

**Figure 4.**
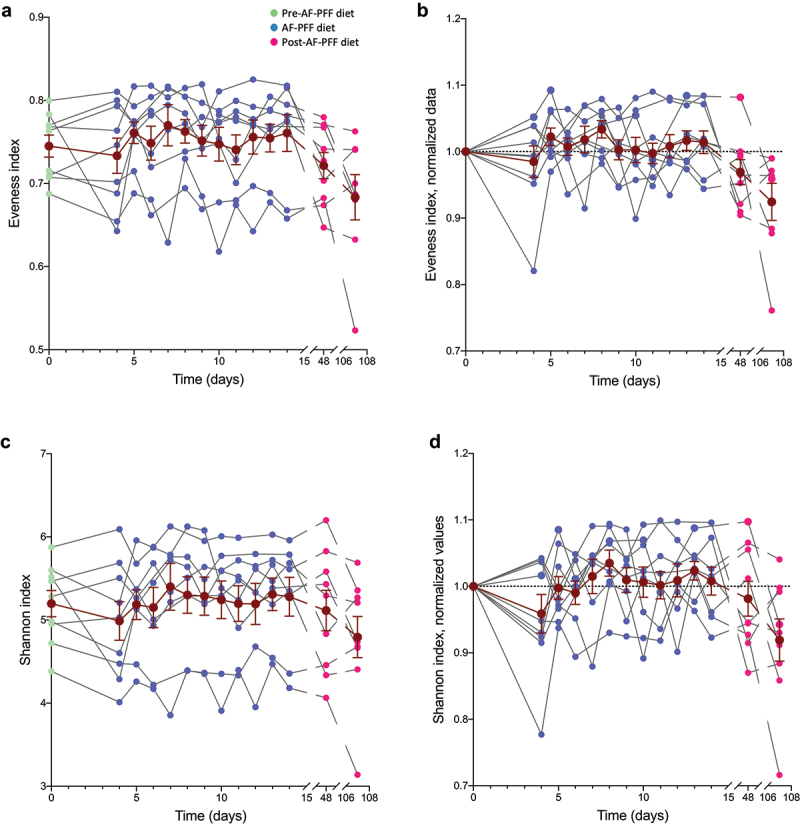
**Diet standardization did not induce changes in individuals’ microbiota alpha diversity**. 9 healthy volunteers were subjected to a controlled diet between day 4 and day 14. (a-b) Evenness alpha diversity matrix throughout the study assessed following 16S rRNA gene sequencing (a), with data in (b) being normalized to 1 for day 0 samples in order to normalize for pre-intervention inter-individual variations. (c-d) Shannon alpha diversity matrix throughout the study assessed following 16S rRNA gene sequencing (c), with data in (d) being normalized to 1 for day 0 samples in order to normalize for pre-intervention inter-individual variations. The dark brown line represents the mean ± SEM and significant was assessed using one-way ANOVA with repeated measures with a Dunnett posttest.

**Figure 5.**
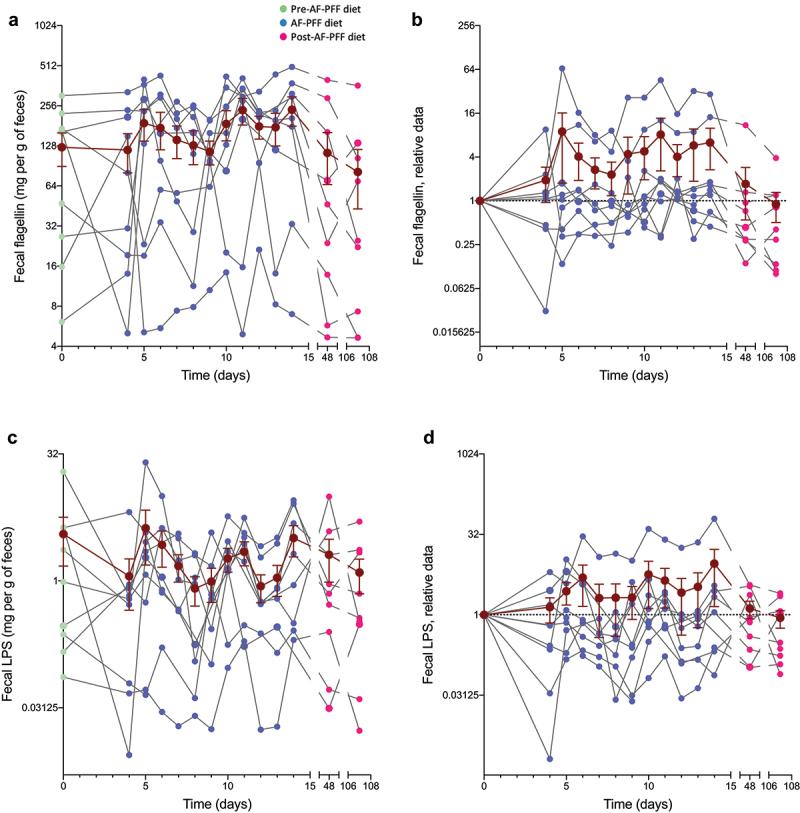
**Microbiota pro-inflammatory potential variations following diet standardization is highly participant dependent**. 9 healthy volunteers were subjected to a controlled diet between day 4 and day 14. (a-b) Fecal level of bioactive flagellin throughout the study measured with HEK-TLR5 reporter cells, with data being expressed in mg per g of feces (a), with data in (b) being normalized to 1 for day 0 samples in order to normalize for pre-intervention inter-individual variations. (c-d) Fecal level of bioactive lipopolysaccharide throughout the study measured with HEK-TLR4 reporter cells, with data being expressed in mg per g of feces (c), with data in (d) being normalized to 1 for day 0 samples in order to normalize for pre-intervention inter-individual variations. The dark brown line represents the mean ± SEM and significant was assessed using one-way ANOVA with repeated measures with a Dunnett posttest.

To further investigate the effects of an AF-PFF diet feeding on participant’s microbiota, we next investigated the Shannon and Eveness indices of alpha diversity, i.e. species richness. We did not observe any alterations in these parameters throughout the study, suggesting that AF-PFF diet is not sufficient, at least during such a short period of exposure and when looking at the average, to significantly impact this microbiota characteristic (Figure 4). As presented in the un-normalized panels **A** and **C**, participant alpha diversity indexes were pretty stable between each phase of our trial, while subtle effects were observed for select participants, suggesting a relatively strong resilience in fecal microbiota richness over time (Figure 4). We next investigated the impact of a controlled homogenous diet on fecal levels of pro-inflammatory molecules lipopolysaccharide (LPS) and flagellin, expressed by numerous microbiota members and previously reported to be a proxy of microbiota pro-inflammatory potential.^12^ Analogous to other parameters measured, levels of fecal flagellin and LPS were highly heterogenous basally and in response to AF-PFF (Figure 5). Accordingly, on average, fecal flagellin and LPS were not significantly impacted by AF-PFF but exhibited a trend of being elevated during the early AF-PFF period, thus paralleling observations re fecal bacterial density. Furthermore, we observed a trend of reduced intra-individual day-to-day variance in fecal LPS and flagellin during the AF-PFF period that did not reach statistical significance (p = .11 and 0.15, **Fig. S5**) but nonetheless accorded with the reduced intra-individual variance in microbiota composition observed during this period.

### Host response heterogeneity to AF-PFF diet

**Figure 6.**
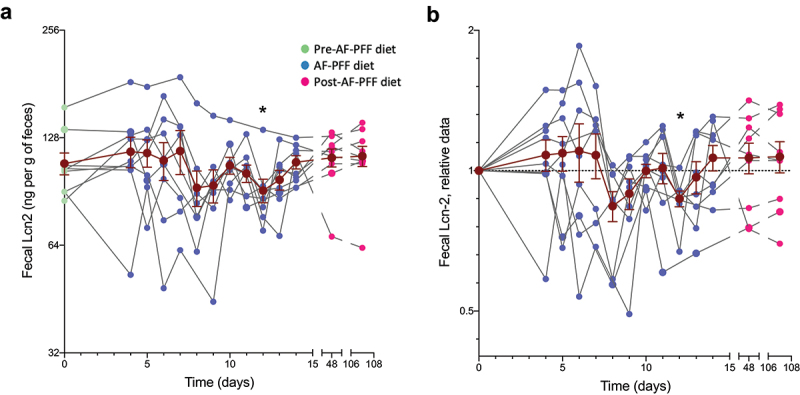
**Host response to a controlled homogenous diet is heterogeneous and individual specific**. 9 healthy volunteers were subjected to a controlled diet between day 4 and day 14. (a-b) Fecal level of the inflammatory marker Lipocalin-2 from day 0 to subsequent days, expressed in ng per g of feces (a), with data in (b) being normalized to 1 for day 0 samples in order to normalize for pre-intervention inter-individual variations. The dark brown line represents the mean ± SEM. Fecal level of bioactive flagellin post-AF-PFF diet (day 14) plotted versus fecal Lipocalin-2 level post-AF-PFF diet (day 14). Significant was assessed using one-way ANOVA with repeated measures with a Dunnett posttest. (*p ≤ 0,05).

Lastly, we investigated impacts of AF-PFF feeding on a host marker of intestinal inflammation, namely fecal lipocalin-2.^13^ Quantification of this parameter in the longitudinally collected fecal samples indicated that diet standardization under an AF-PFF was not sufficient to impact inflammatory tone although select participants harbored a relatively stable decrease in fecal lipocalin-2 level during the AF-PFF phase (Figure 6. a, b). Looking at levels of lipocalin-2, flagellin and LPS within each participant also revealed a highly heterogenous response to AF-PFF (**Fig. S6**). Thus, AF-PFF does not, by itself, significantly impact microbiota or host parameters relating to inflammation.

## Discussion

During the last three decades, accumulating evidence has revealed a central role played by the intestinal microbiota in determining health and disease.^14^ Roles of this complex microbial community include promoting maturation of the intestinal immune system and protection against enteric pathogens. Furthermore, alteration in microbiota composition and/or function plays a role in the promotion of numerous diseases with an inflammatory component, such as inflammatory bowel diseases and metabolic syndrome.^1,2,15,16^ In addition to disease-associated alterations in microbiota, there is a wide range in the variance of microbiota among healthy individuals. While much effort is directed toward understanding microbiota dysbiosis, the mechanisms by which the stability of a microbiota is maintained within healthy individuals and how they become altered in disease remain poorly defined. Studies in mice suggest that numerous genetic and environmental factors are involved in shaping the composition of the intestinal microbiota, among which diet appears to play a key role, perhaps reflecting that food ingested is having a daily impact on this complex ecosystem equilibrium. By contrast, the extent to which diet alters the human microbiota composition stability and, concomitantly, perturbation in response to disease, is much less clear.

This study sought to take advantage of the control arm of the recently performed FRESH study^9^ in order to investigate the extent to which diet normalization with an additive free and processed food-free diet might impact inter- and/or intra-individual microbiota variation. We observed that standardizing diet with an AF-PFF diet across healthy adults did not reduce inter-individual differences in microbiota composition but reduced day-to-day variations within individuals, supporting the notion that diet heterogeneity contributes to variations in microbiota composition. Such decreased intra-individual microbiota variations were relatively rapid to arise (7 days), while their stability through time remains to be investigated since no samples were collected in the days directly following the cessation of the AF-PFF diet. Moreover, the inability of AF-PFF to reduce inter-subject variance argues that such variance is not the sole consequence of food additives, at least over the short time period of our study, aligning with previous observations of relatively high inter-subject differences in microbiota composition.^7,17^ Yet, given previous observations that long-term dietary habits associate with broad taxonomic differences,^7^ together with our observation that additives withdrawal is sufficient to induce stark increase in health-associated microbiota members (*Bifidobacteriales* and *Verrucomicrobiales* orders) in select individuals, we suggest that investigation of whether similar AF-PFF might reduce inter-individual differences over a longer period are warranted. In addition to diet standardization effect, hospitalization of the participant during the AF-PFF period enables better sample handling and conservation in a way that could impact the relative abundance of select microbiota members. This may artificially decrease experimentally induced variations and thus could play a role in the observed reduced intra-individual variations during the AF-PFF diet.

Importantly, the observation that intra-subject variability was reduced during the FRESH study generally supports the notion that diet impacts microbiota. Similar results were seen by Sonnenberg et al.^18^ Using a 7-day homogenous diet, similar reduction of intrapersonal microbiota composition variation, unchanged species richness and diversity, as well as individual variation in fecal metabolites were observed, in accordance with observation made with our AF-PFF diet approach. Concordance of these results under two homogenous but different diets, both in composition and duration, emphasize the ability of diet homogenization to dampen day-to-day microbiota variance. Homogenous diet consumption by participants in Sonnenberg’s study also induced a small but significant decrease in interpersonal variation during homogenous diet phase, in contradiction with the unchanged variation level observed during AF-PFF. We note that our observation of the inability of AF-PFF to reduce inter-subject variance in microbiota composition align with previous observations made in a seminal controlled-feeding study.^7^ These contradictory results might suggest that higher participant number is needed to decipher such small effect of diet homogenization on inter-personal variations. Another possibility is that, given that food additives may have an effect on gut microbiota composition, homogenization of food additive intake in this past study is driving most of the reduction in inter-individual variability observed, while the AF-PFF diet conserved the inter-individual variability by preventing the exposure of all participants to a similar diet-driven stressors.

Altogether, these findings support the notion that diet standardization can eventually optimize the power of investigation into the ability of a given compound/additive to modulate the intestinal microbiota composition and function. Indeed, while this remains to be tested, day-to-day variance driven by the impact of undefined dietary factors on gut microbiota composition could potentially mask the effect of the dietary factor being studied.

## Methods

### Study design

Data used in this addendum originates from a randomized, controlled-feeding study performed in the University of Pennsylvania’s Center for Human Phenomic Science (CHPS) and registered at https://ClinicalTrials.gov as trial no. NCT03440229.^9^ The first 3 days of the study were as an outpatient followed by 11 days as an inpatient. Once admitted to the CHPS unit, participants were not allowed to leave the unit unless accompanied by study staff. The study included nine healthy volunteers that were submitted to an additive-free standardized diet for 14 days. Full study information are described in Chassaing et al.^9^

### Diet

All food was prepared within the CHPS metabolic kitchen without emulsifiers. All participants followed the same Western-style diet (the only difference being portion size). The macronutrient percentages of calories for the study diet were 55% carbohydrate, 30% fat, and 15% protein. The diet provided is considered healthy with a Healthy Eating Index score of 75.^19–21^ The diet was composed of two menus that were consumed on alternating days. Water, black coffee, and plain tea were provided as desired. Participants had access to additional servings of food beyond the meals provided; however, the entire serving of the previous meal must have been consumed to receive additional servings.

### Sample collection

Stool samples were collected without preservatives or stabilizers before starting the outpatient diet, daily during the inpatient stay, and at 1 and 3 months after discharge. The first stool sample of the day was aliquoted and frozen at −80°C. All other stool samples during the inpatient stay were weighed and then discarded. On inpatient days 4 and 14, each participant underwent a sigmoidoscopy to obtain biopsies from the area of approximately 15 cm from the anal verge, which correlates with approximately the rectosigmoid junction. No bowel preparation was utilized prior to the sigmoidoscopy. Biopsy samples were placed in Carnoy solution for non-denaturing confocal microscopy.

### Microbiota analysis by 16S rRNA gene sequencing using Illumina technology

16S rRNA gene amplification and sequencing utilized the Illumina MiSeq technology following the protocol of Earth Microbiome Project with their modifications to the MOBIO PowerSoil DNA Isolation Kit procedure for extracting DNA http://www.earthmicrobiome.org/emp-standard-protocols.^2,3^ Bulk DNA was extracted from frozen feces using a PowerFecal-HT kit from Qiagen with mechanical disruption (bead-beating). The 16S rRNA genes, region V4, were PCR amplified from each sample using a composite forward primer and a reverse primer containing a unique 12-base barcode, designed using the Golay error-correcting scheme, which was used to tag PCR products from respective samples.^3^ We used the forward primer 515 F 5’- *AATGATACGGCGACCACCGAGATCTACACGCT*XXXXXXXXXXXX**TATGGTAATT*GT***GTGYCAGCMGCCGCGGTAA-3’: the italicized sequence is the 5’ Illumina adapter, the 12 × Sequence is the Golay barcode, the bold sequence is the primer pad, the italicized and bold sequence is the primer linker and the underlined sequence is the conserved bacterial primer 515 F. The reverse primer 806 R used was 5’-*CAAGCAGAAGACGGCATACGAGAT***AGTCAGCCAG*CC***GGACTACNVGGGTWTCTAAT-3’: the italicized sequence is the 3’ reverse complement sequence of Illumina adapter, the bold sequence is the primer pad, the italicized and bold sequence is the primer linker and the underlined sequence is the conserved bacterial primer 806 R. PCR reactions consisted of Hot Master PCR mix (Quantabio, Beverly, MA, USA), 0.2 μM of each primer, 10–100 ng template, and reaction conditions were 3 min at 95°C, followed by 30 cycles of 45 s at 95°C, 60 s at 50°C and 90 s at 72°C on a Biorad thermocycler. PCR products were quantified using Quant-iT PicoGreen dsDNA assay, a master DNA pool was generated from the purified products in equimolar ratios and subsequently purified with Ampure magnetic purification beads (Agencourt, Brea, CA, USA). The pooled product was quantified using Quant-iT PicoGreen dsDNA assay and then sequenced using an Illumina MiSeq sequencer (paired-end reads, 2 × 250 bp) at Cornell University, Ithaca, NY.

### 16S rRNA gene sequence analysis

16S rRNA sequences were analyzed using QIIME2 – version 2019^22^ Sequences were demultiplexed and quality filtered using Dada2 method^23^ with QIIME2 default parameters in order to detect and correct Illumina amplicon sequence data, and a table of Qiime 2 sequence variants (SVs) was generated. A tree was next generated, using the align-to-tree-mafft-fasttree command, for phylogenetic diversity analyses, and alpha and beta diversity analyses were computed using the core-metrics-phylogenetic command. In order to normalize for inter-individual variations in microbiota composition, day 1 data were normalized at 1 for every SV identified, and the data for the other days were expressed, for each individual patient, as relative values compared to day 1 data. Principal coordinate analysis (PCoA) of the Jaccard metric was visualized to assess the variation between samples (beta diversity). For taxonomy analysis, taxonomy was assigned to SVs with a 99% threshold of pairwise identity to the Greengenes reference database 13_8.^24^ ASVs significantly impacted by diet standardization were identified using MaAsLin2 R package, R software version 4.1.2. Unprocessed sequencing data are deposited in the European Nucleotide Archive under accession number PRJEB55423.

### Bacterial density quantification by 16S rRNA qPCR

Extracted DNAs were diluted 1/10 with sterile DNA-free water and amplified by quantitative PCR using the 16S V4-specific primers 515 F 5’-GTGYCAGCMGCCGCGGTAA-3’ and 806 R 5’-GGACTACNVGGGTWTCTAAT-3’ or using the AIEC LF82 PTM specific primers PTM-F 5’- CCATTCATGCAGCAGCTCTTT −3’ and PTM-R 5’- ATCGGACAACATTAGCGGTGT −3’ on a LightCycler 480 (Roche) using QuantiFast SYBR® Green PCR Kit (Qiagen). Amplification of a single expected PCR product was confirmed by electrophoresis on a 2% agarose gel, and data are expressed as relative values normalized with feces weight used for DNA extraction.

### Fecal flagellin and lipopolysaccharide load quantification

Levels of fecal bioactive flagellin and lipopolysaccharide (LPS) were quantified as previously described^25^ using human embryonic kidney (HEK)-Blue-mTLR5 and HEK-BluemTLR4 cells, respectively (Invivogen, San Diego, CA, USA). Fecal material was resuspended in PBS to a final concentration of 100 mg/mL and homogenized for 10 s using a Mini-Beadbeater-24 without the addition of beads to avoid bacteria disruption. Samples were then centrifuged at 8000 g for 2 min, and the resulting supernatant was serially diluted and applied on mammalian cells. Purified E. coli flagellin and LPS (Sigma-Aldrich) were used for standard curve determination using HEK-Blue-mTLR5 and HEK-Blue-mTLR4 cells, respectively. After 24 h of stimulation, the cell culture supernatant was applied to QUANTI-Blue medium (Invivogen), and the alkaline phosphatase activity was measured at 620 nm after 30 min.

### Metabolomic analysis of stool samples

Stool sample preparation for NMR was performed as previously described^1,26^H NMR spectra were acquired on a Bruker Avance NEO 600 MHz spectrometer equipped with an inverse cryogenic probe (Bruker Biospin, Germany) at 298 K. A typical 1D NMR spectrum named NOESYPR1D was acquired for each sample. The metabolites were assigned on the basis of published results^27^ and confirmed with a series of 2D NMR spectra. All^1^H NMR spectra were adjusted for phase and baseline using Chenomx (Chenomx Inc, Canada). The chemical shift of ^1^H NMR spectra was referenced to sodium 3-trimethylsilyl [2,2,3,3-d4] propionate (TSP) at δ 0.00. The relative contents of metabolites were calculated by normalizing to the total sum of the spectral integrals. The quantification of metabolites in stool was calculated by NMR peak area against TSP using Chenomx.

### Quantification of fecal lipocalin-2 (Lcn-2) by ELISA

For quantification of fecal Lcn-2 by ELISA, frozen fecal samples were reconstituted in PBS containing 0.1% Tween 20 to a final concentration of 100 mg/mL and vortexed for 20 min to get a homogenous fecal suspension.^13^ These samples were then centrifuged for 10 min at 14 000 g and 4°C. Clear supernatants were collected and stored at **−**20°C until analysis. Lcn-2 levels were estimated in the supernatants using Duoset Human Lcn-2 ELISA kit (R&D Systems, Minneapolis, MN, USA) using the colorimetric peroxidase substrate tetramethylbenzidine, and optical density (OD) was read at 450 nm (Versamax microplate reader).

### Statistical analysis

Significance was determined using one-way ANOVA with or without repeated measures, corrected for multiple comparisons with a Dunnett or Sidak posttest, respectively. Significance of data that did not respect normality and homoscedasticity postulates was tested using Kruskal-Wallis corrected for multiple comparisons with a Dunn’s test and Brown-Forsythe and Welch ANOVA corrected for multiple comparisons with a Dunnett test, respectively. Differences were noted as significant at P ≤ .05.

## Supplementary Material

Supplemental Material
