# Supplementary material for: Diet standardization reduces intra-individual microbiome variation
Source: Gut Microbes. 2022 Nov 25;14(1):2149047. doi: 10.1080/19490976.2022.2149047 (PMC9704386; doi:10.1080/19490976.2022.2149047)
Supplement: Supplemental Material [file KGMI_A_2149047_SM6428.pptx]

## Slide 1
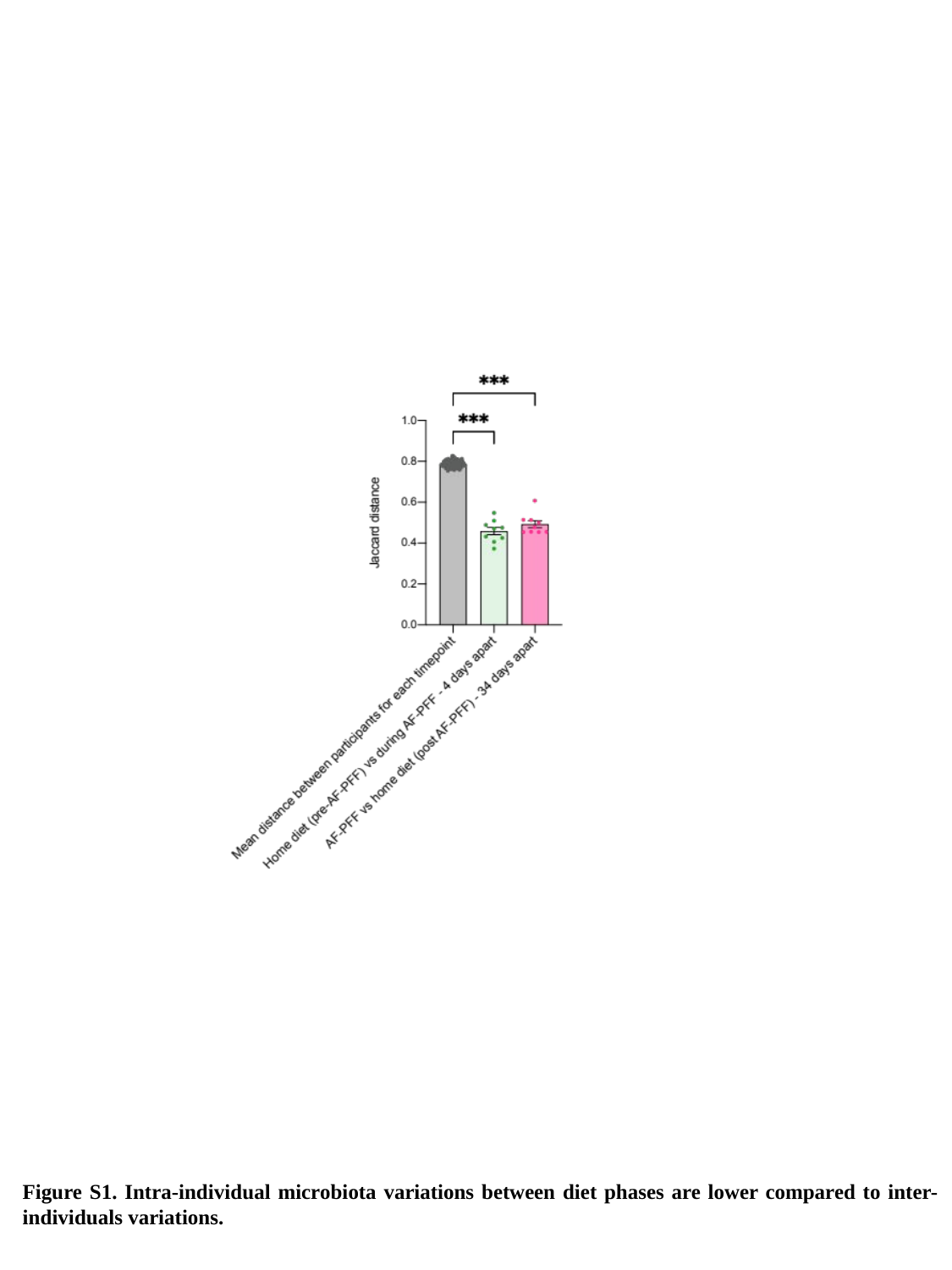

Figure S1. Intra-individual microbiota variations between diet phases are lower compared to inter-individuals variations.

## Slide 2
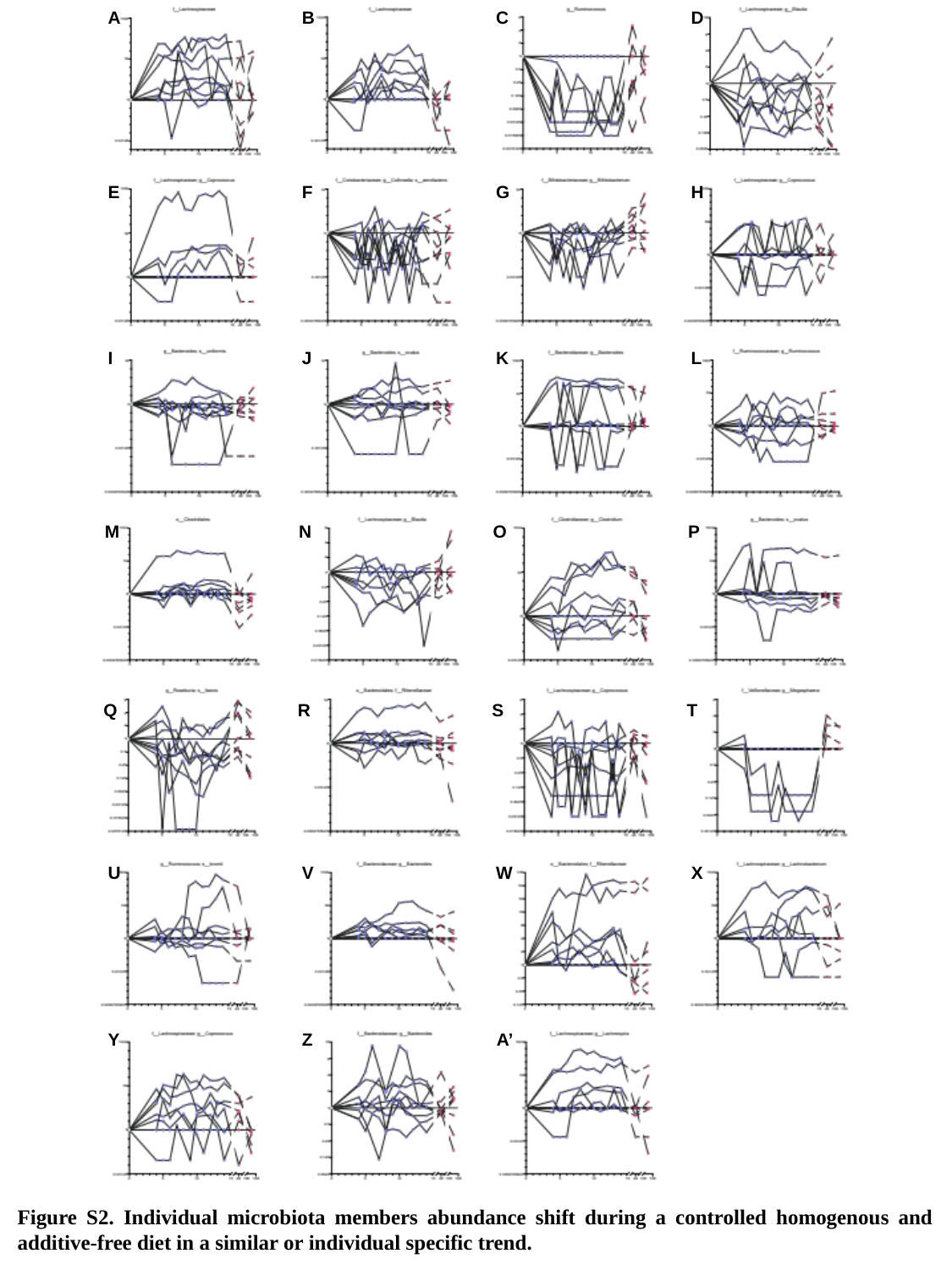

A
B
C
D
E
F
G
H
I
J
K
L
M
N
O
P
Q
R
S
T
U
V
W
X
Y
Z
A’
Figure S2. Individual microbiota members abundance shift during a controlled homogenous and additive-free diet in a similar or individual specific trend.

## Slide 3
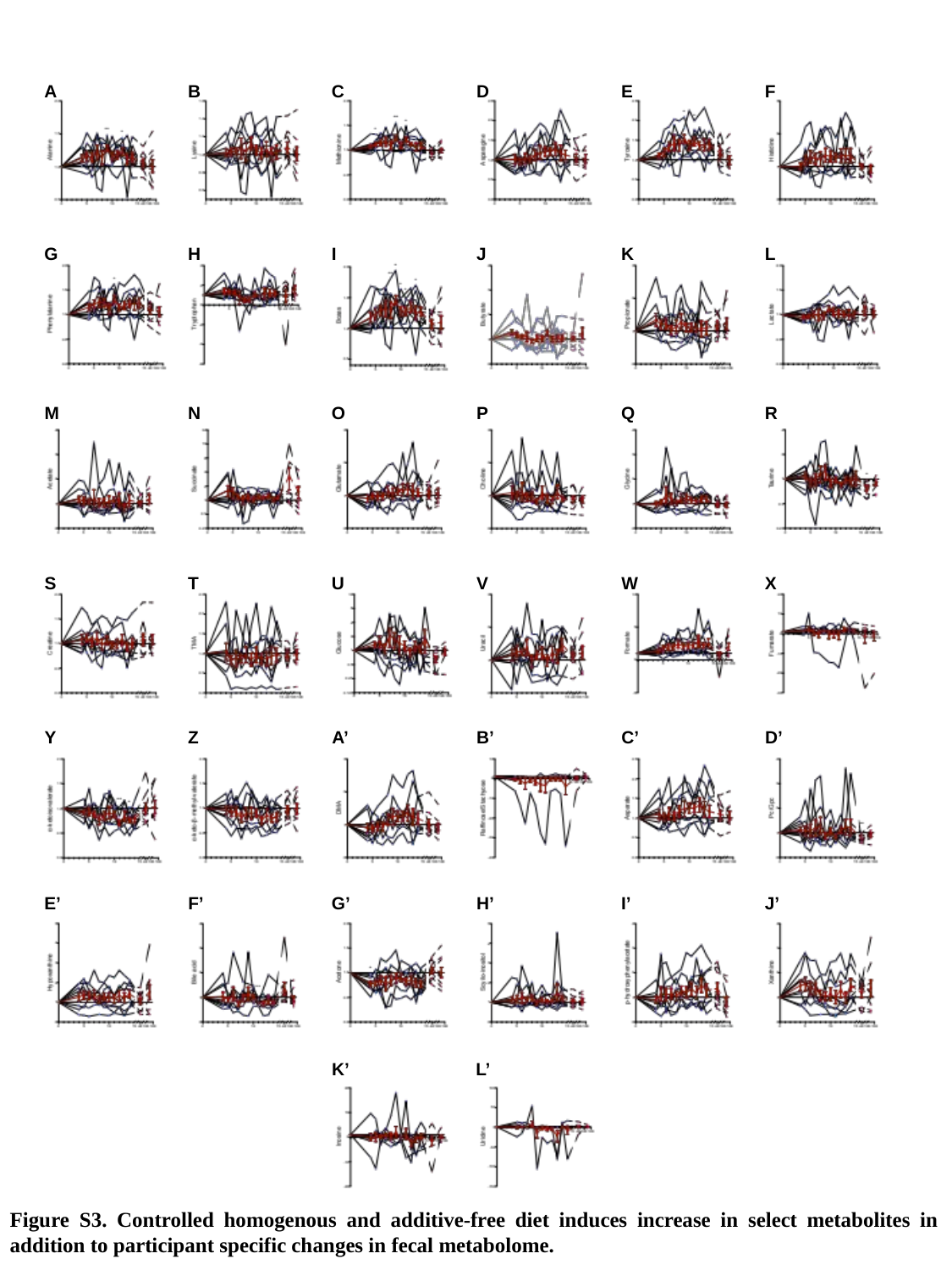

A
B
C
D
E
F
G
H
I
J
K
L
M
N
O
P
Q
R
S
T
U
V
W
X
Y
Z
A’
B’
C’
D’
E’
F’
G’
H’
I’
J’
K’
L’
Figure S3. Controlled homogenous and additive-free diet induces increase in select metabolites in addition to participant specific changes in fecal metabolome.

## Slide 4
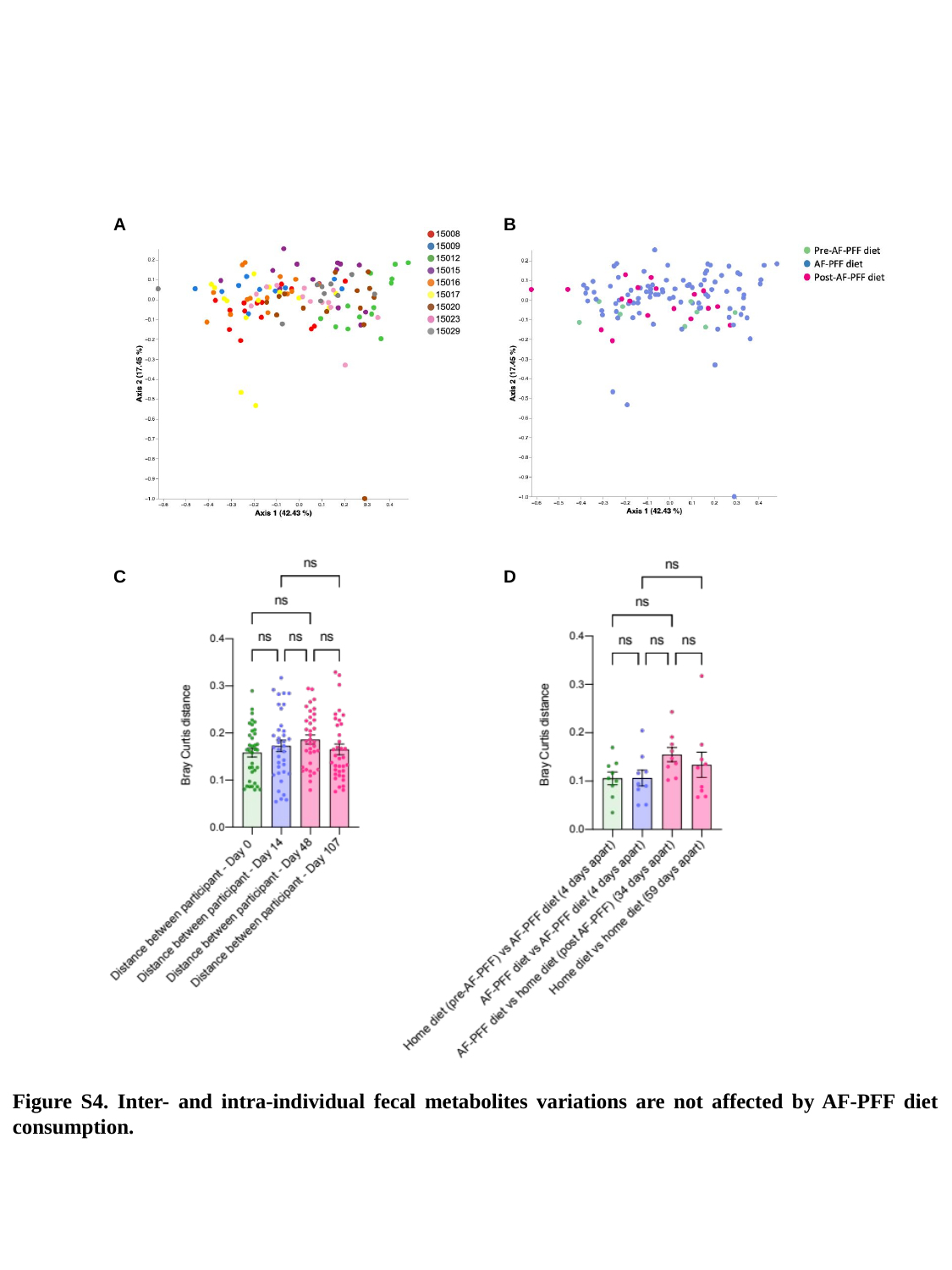

B
A
C
D
Figure S4. Inter- and intra-individual fecal metabolites variations are not affected by AF-PFF diet consumption.

## Slide 5
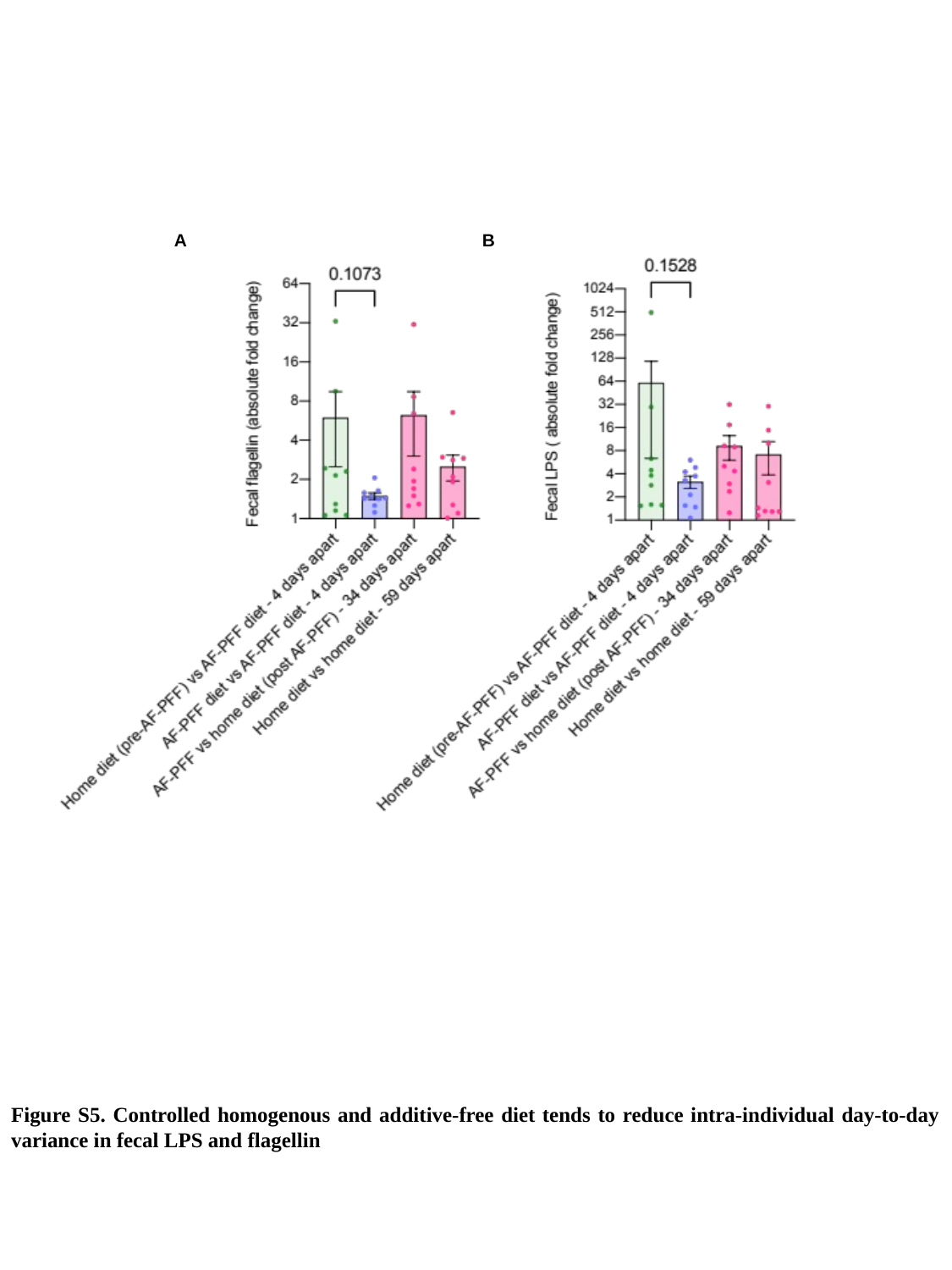

A
B
Figure S5. Controlled homogenous and additive-free diet tends to reduce intra-individual day-to-day variance in fecal LPS and flagellin

## Slide 6
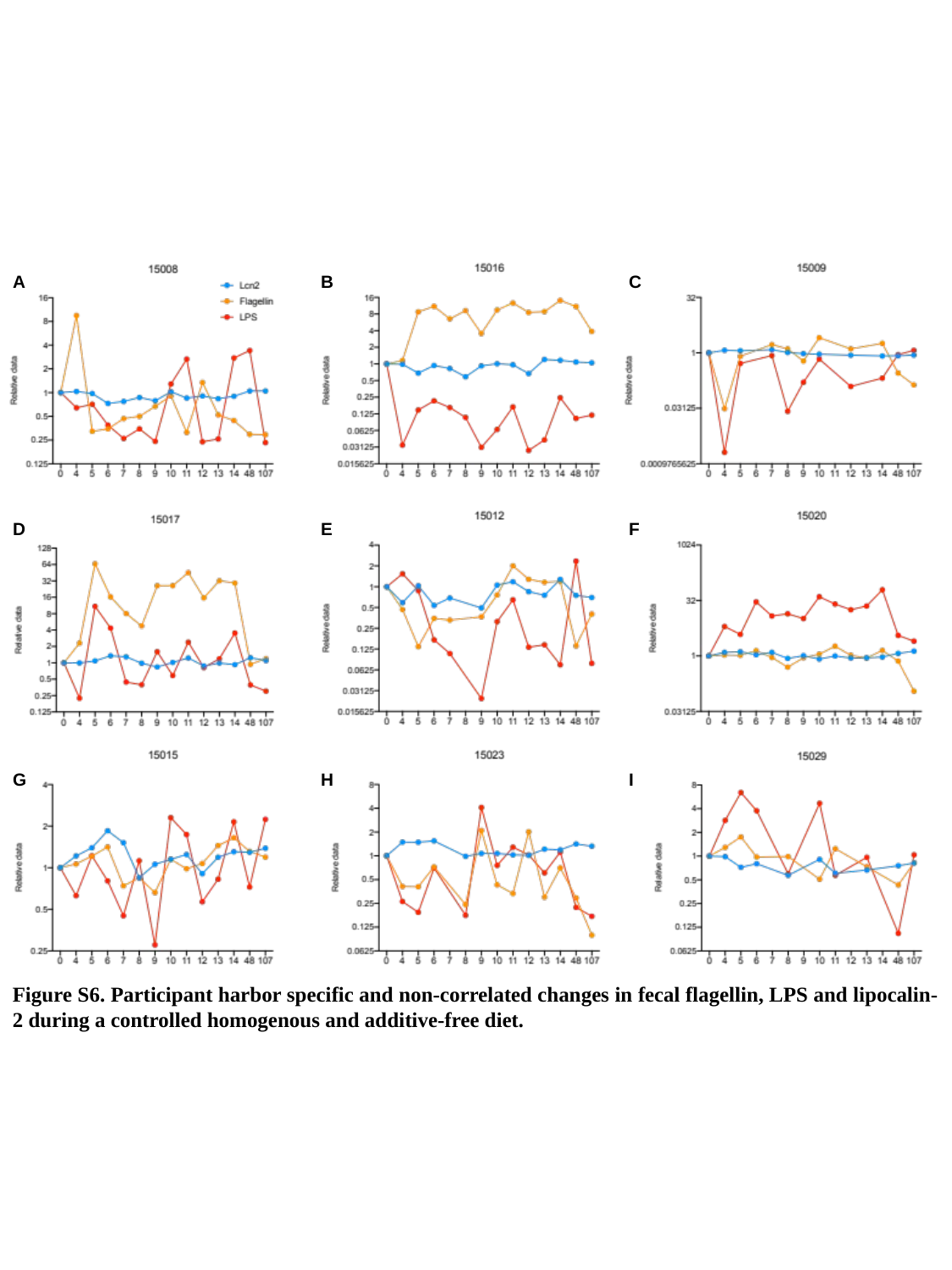

C
A
B
F
D
E
I
G
H
Figure S6. Participant harbor specific and non-correlated changes in fecal flagellin, LPS and lipocalin-2 during a controlled homogenous and additive-free diet.
